# A rare case of clear cell sarcoma-like/malignant gastrointestinal neuroectodermal tumor in the pancreas: case report and literature review

**DOI:** 10.3389/fmed.2026.1768345

**Published:** 2026-03-19

**Authors:** Jing Huang, Lu Zhou, Xiao-Yu Chen, Yu-Zhen Huang, Ru-Wei Mo, Li-Xia Zeng, Sheng-Ming Wu, Wen-Qi Luo

**Affiliations:** Department of Pathology, Guangxi Medical University Cancer Hospital, Nanning, China

**Keywords:** fluorescence *in situ* hybridization, gastrointestinal clear cell sarcoma-like tumor, malignant gastrointestinal neuroectodermal tumor, pancreas, soft tissue neoplasm

## Abstract

Malignant gastrointestinal neuroectodermal tumor (M-GNET), also known as clear cell sarcoma-like tumor of the gastrointestinal tract (CCSLTGT) or clear cell sarcoma-like tumor of the gastrointestinal tract with osteoclast-like giant cells, is a rare malignant tumor that typically arises in the gastrointestinal tract. It demonstrates primitive neural or neuroectodermal differentiation but lacks melanocytic features. M-GNET is closely related to clear cell sarcoma of soft tissue (CCSST), sharing highly overlapping morphological features and molecular genetic characteristics, particularly *EWSR1-ATF1* gene fusion and, more rarely, *EWSR1-CREB1* gene fusion. Most M-GNETs occur in the lower gastrointestinal tract, while only a few cases have been reported in the upper gastrointestinal tract and outside the digestive tract. We present the primary M-GNET in the pancreas, confirmed by molecular fluorescence *in situ* hybridization (FISH) detection of *EWSR1-ATF1* gene fusion. This article will summarize the clinicopathological features and differential diagnosis and review the relevant literature.

## Introduction

Malignant gastrointestinal neuroectodermal tumor (M-GNET)/clear cell sarcoma-like tumor of the gastrointestinal tract (CCSLTGT) and clear cell sarcoma of soft tissue (CCSST) are exceptionally rare and closely related malignant mesenchymal neoplasms, characterized by varying degrees of melanocytic differentiation. In the 2019 fifth edition of the WHO Classification of Tumors of the Digestive System, M-GNET and CCSST were categorized together under the group of undifferentiated tumors. CCSST was first reported by Enzinger in 1965 as a rare sarcoma occurring in the tendons and aponeuroses of the lower extremities in young individuals (1). Although its morphological features resemble those of soft tissue melanoma, CCSST differs in clinical, pathological, and genetic characteristics, and is considered a distinct entity separate from malignant melanoma. In 1993, Ekfors first reported a case of CCSST arising in the duodenum (2). Zambrano et al. introduced the term “clear cell sarcoma-like tumor of the gastrointestinal tract (CCSLT)” to describe a neoplasm sharing certain features with CCSST but is enriched with osteoclast-like giant cells, exhibits more extensive histological variations, and lacks expression of melanocytic markers (3). Bridge et al. identified the t (12;22) (q13;q12) translocation resulting in the EWS RNA binding protein 1 gene (*EWSR1*) (22q12)*-*activating transcription factor 1 (*ATF1*) fusion gene in CCSLT (4). This unique chromosomal rearrangement, distinct from that of cutaneous malignant melanoma, established CCSLT as a separate entity. Stockman et al. found ultrastructural evidence of primitive neuroectodermal differentiation in these tumors and proposed the term “malignant gastrointestinal neuroectodermal tumor” (M-GNET) as a more appropriate diagnostic label (5). Some authors, however, prefer the term “clear cell sarcoma-like tumor of the gastrointestinal tract (CCSLTGT).”

Gastrointestinal M-GNET/CCSLTGT typically originates in the muscularis propria, often presenting as an endophytic, solid, lobulated mass measuring 2–15 cm in diameter. It frequently ulcerates, resembling common malignancies, and exhibits a brownish cut surface. Histologically, tumor cells are arranged in solid sheets, nests, pseudopapillary, or pseudoalveolar patterns. The cells are oval, short spindle, or epithelioid in shape, with vesicular chromatin and inconspicuous nucleoli, and their cytoplasm is usually faintly eosinophilic. Clear cytoplasm is observed in only a few cases. Approximately half of the cases contain osteoclast-like giant cells, and the number of mitoses varies. Immunohistochemically, the tumor is positive S100 and SRY-box 10 (SOX10), whereas melanocytic markers such as HMB45, Melan-A, and microphthalmia-associated transcription factor (MiTF) are almost always negative. At the molecular genetic level, t(12;22) (q13;q12) and t(2;22) (q34;q12) translocations generate the *EWSR1-ATF1* and *EWSR1-CREB1* fusion genes.

To date, only 24 well-documented cases of extragastrointestinal M-GNET—confirmed by molecular analysis and lacking melanocytic markers—have been reported (summarized in Table 1). Herein, we present the case of a clear cell sarcoma-like/malignant gastrointestinal neuroectodermal tumor originating in the pancreas, detailing its clinical features, pathological characteristics, differential diagnosis, and prognosis, along with a review of the relevant literature.

**Table 1:** A summary of reported cases of extra-enteric clear cell sarcoma-like/malignant gastrointestinal neuroectodermal tumor.

Case	Reference	Age/Sex	Location/size (cm)	Immunophenotype				Molecular genetic features	Treatment	Follow up (months)	Outcome
				S100	SOX10	HMB45	MelanA				
1	Kraft et al. (18)	82/Female	Tongue/2.0	+	NA	−	−	EWSR1::ATF1	Adjuvant RT	7	AWD
2	Kim et al. (13)	21/Male	Esophagus/3.5	+	+	−	−	EWSR1 rearrangement	Surgery + adjuvant RT	5	ANED
3	Baus et al. (26)	44/Female	Tongue/4.1	+	NA	−	NA	EWSR1 rearrangement	Neoadjuvant CT + surgery + adjuvant RT	6	ANED
4	Song et al. (27)	23/Male	Esophagus/4.5	+	+	NA	−	NA	Surgery	24	ANED
5	Allanson et al. (11)	5/Female	Palate/2.6	+	+	−	−	EWSR1::ATF1	Biopsy	16	AWD
6	Zheng et al. (21)	40/Male	Left bronchus/ 1.5	+	+	−	−	EWSR1::ATF1 and SOX1-OT::KMT2C	Bronchoscopic resection+ CT + adjuvant RT	24	AWD
7	Yang et al. (28)	47/Female	Right thigh/ 6.0	+	+	−	−	EWSR1::ATF1	Surgery	11	ANED
8	Sbaraglia et al. (29)	62/Male	Tongue/ 3.3	+	NA	−	−	EWSR1::CREB1	Surgery + adjuvant RT	4	ANED
9	Li et al. (22)	62/Male	Right atrium and right ventricle/ 3.0 and 4.7	+	+	−	NA	EWSR1::ATF1	Surgery	20	DOD
10	Kuo et al. (30)	27/Male	Larynx/4.5	+	+	−	−	EWSR1::ATF1	Surgery	8	ANED
11	Kuo et al. (30)	56/Male	Intracranial (skull base)	+	+	−	−	EWSR1::ATF1	Surgery	3	AWD
12	Jiang et al. (20)	26/Female	Esophagus/6	+	+	−	NA	EWSR1::CREB1	Surgery + adjuvant RT + Targeted Therapy	22	AWD
13	Sugimoto et al. (31)	38/Female	Retroperitoneum/ 7.0	+	+	−	−	EWSR1::CREB1	Surgery	8	AWD
14	Ulici et al. (19)	70/Female	Chest wall/ NA	+	+	−	−	EWSR1::CREB1	Surgery	25	DOD
15	Ulici et al. (19)	36/Male	Bladder/ NA	+	+	−	−	EWSR1::ATF1	Surgery + adjuvant RT + CT	45	AWD
16	Ulici et al. (19)	14/Male	Right neck/ 3.7 cm	+	+	−	−	EWSR1::ATF1	Surgery + adjuvant RT	11	AWD
17	Ulici et al. (19)	29/Female	Right shoulder/NA	+	NA	−	−	EWSR1::ATF1	NA	12	DOD
18	Ulici et al. (19)	30/Male	Right neck/ NA	+	+	−	−	EWSR1::ATF1	Surgery + adjuvant RT	70	ANED
19	Ulici et al. (19)	48/Female	Left buttock/ NA	+	+	−	−	EWSR1::PBX1	Surgery	53	ANED
20	Ulici et al. (19)	48/Female	Right neck/ 5.5	+	+	−	−	EWSR1::ATF1	Surgery	10	ANED
21	Ulici et al. (19)	30/Female	Liver and falciform ligament/ 10	+	+	−	−	EWSR1::CREB1	Surgery	11	DOD
22	Ulici et al. (19)	29/Female	Left orbit / NA	+	+	NA	NA	EWSR1::ATF1	Surgery + adjuvant RT	64	DOD
23	Ulici et al. (19)	45/Female	Tongue and parapharyngeal space/ >5	+	+	NA	−	EWSR1::CREB1	Surgery	47	ANED
24	Ulici et al. (19)	40/Female	Left gluteus	+	+	−	−	EWSR1::ATF1	Biopsy	NA	NA

A summary of reported cases of extra-enteric clear cell sarcoma-like/malignant gastrointestinal neuroectodermal tumor.

NA, not available; RT, radiotherapy; CT, chemotherapy; AWD, alive with disease; ANED, alive with no evidence of disease.

The case was initially diagnosed as CCS, but the described features are more consistent with GNET.

## Case report

**Figure 1:**
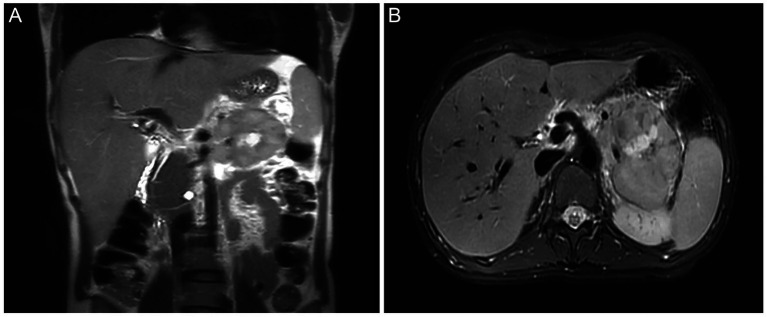
Radiological images. MRI scans showed an occupying lesion involving the tail of the pancreas (**A**: coronal view; **B**: axial view).

**Figure 2:**
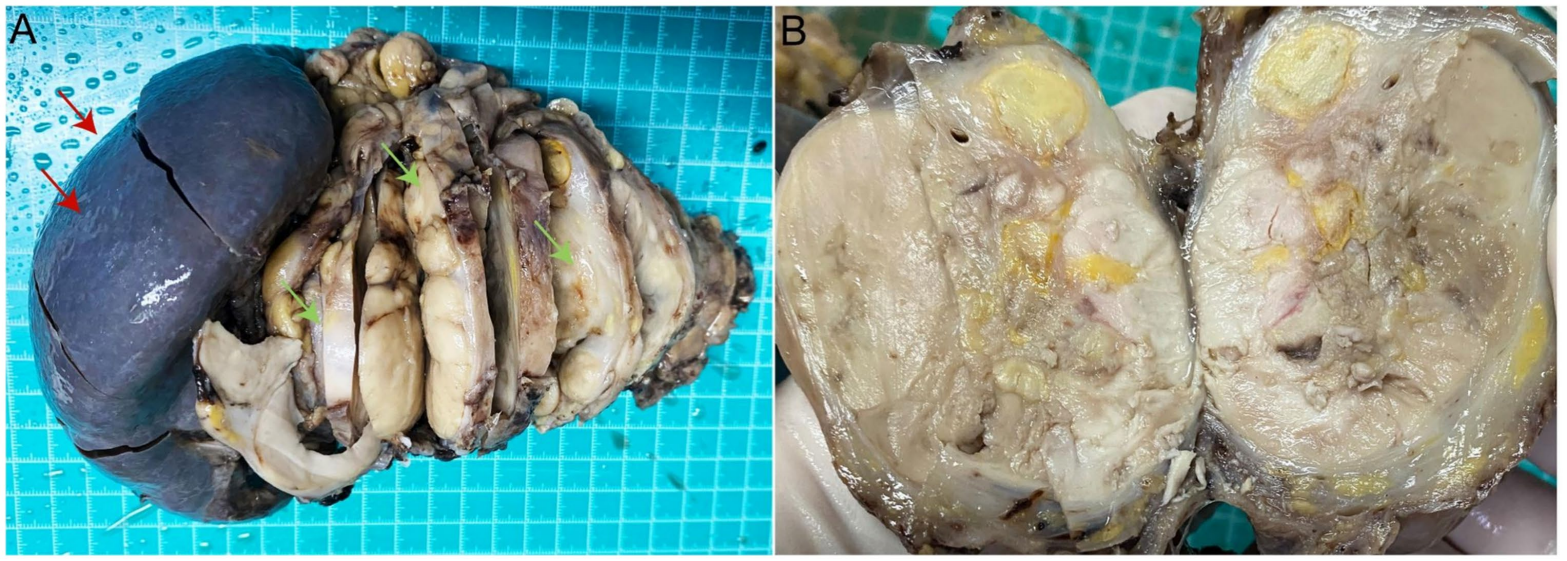
Gross picture. **(A)** A mass of 13 cm × 7 cm × 5 cm is located at the body and tail of the pancreas (green arrow), adjacent to the spleen (red arrow). **(B)** Horizontal section, the tumor section is yellow-white, with multiple nodules and relatively clear boundaries.

**Figure 3:**
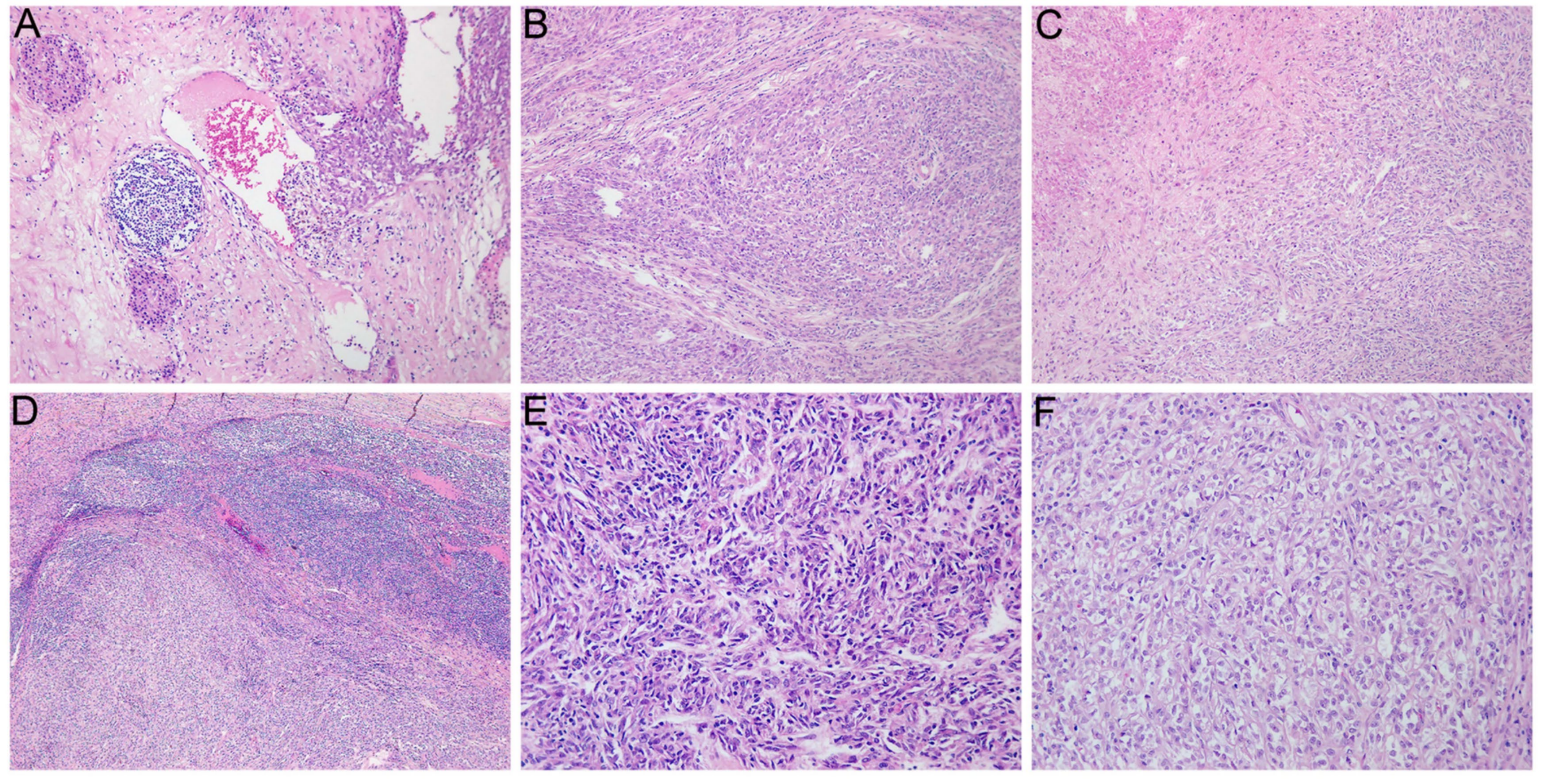
Histological findings. **(A)** The tumor is located within the pancreatic parenchyma, with fibrosis and atrophy of the surrounding pancreatic tissue (×100). **(B)** Fibrous separation was notable (×100). **(C)** Focal areas necrosis (×100). **(D)** Lymph node metastasis (×100). **(E)** The neoplastic cells are spindle-shaped or oval, proliferated diffusely in the pattern of nests and trabeculae with abundant interstitial blood vessels (×200). **(F)** The tumor cells are epithelioid or oval-shaped, with small nucleoli, clear cytoplasm and slightly irregular outlines (×200).

**Figure 4:**
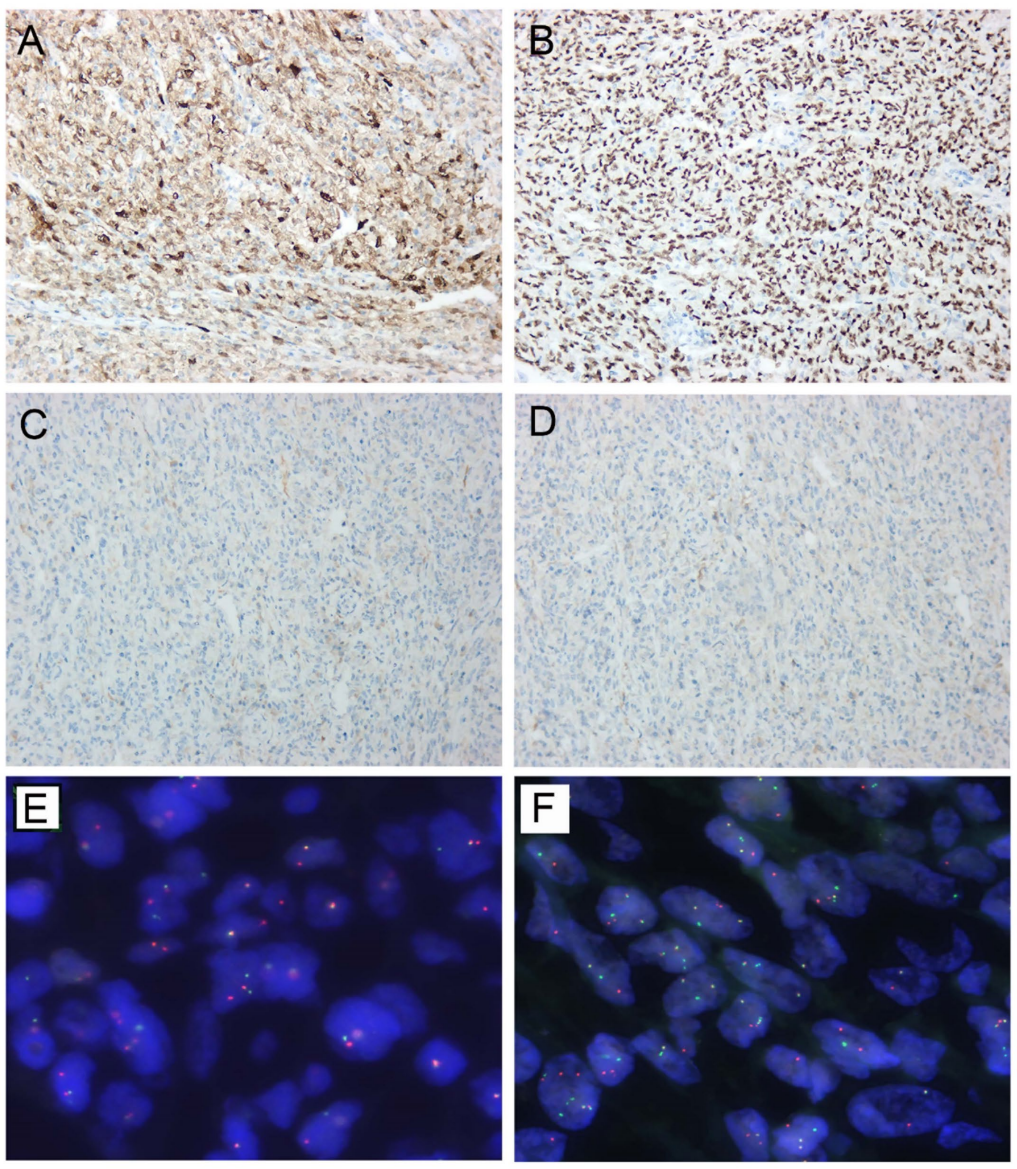
Immunostaining and FISH analysis. Immunohistochemistry showed **(A)** diffusely positive for S100 **(A)** and SOX10 **(B)**, while negative for HMB45 **(C)** and Melan-A **(D)** (all ×200). **(E)** Break-apart fluorescence *in situ* hybridization (FISH) for *EWSR1*(22q12) showed a split signal pattern in most of the tumor cells. **(F)** *EWSR1-ATF1* fusion by FISH analysis showed two fusion signals (indicated by the white arrows), and a separate red and green signal.

A 29-year-old female presented in September 2023 with recurrent postpartum-fever lasting one month. A CT scan performed at another hospital revealed an irregular iso−/hypodense lesion in the body and tail of the pancreas. The patient was then transferred to our institution. MRI demonstrated an irregular abnormal signal mass in the pancreatic body and tail with indistinct margins (Figures 1A,B). She underwent a subtotal pancreatectomy and splenectomy. Gross examination revealed a large tumor within the pancreatic tissue, measuring 13 cm × 7 cm × 5 cm. The main bulk of the tumor was located in the tail of the pancreas, partially encapsulated. The cut surface was grayish-white, solid, moderately firm, and exhibited a multinodular, confluent appearance with focal areas of necrosis (Figures 2A,B). Microscopically, the tumor exhibited multiple confluent nodules. Within each nodule, tumor cells were arranged in trabecular, cord-like, and nest-like patterns, with extensive central necrosis. Most cells were oval or spindle-shaped, although, in certain regions, the cells appeared epithelioid or round, with moderate, clear cytoplasm and prominent nucleoli. Pathologic mitotic figures numbered 16 per 10 high-power fields (Figure 3). One peripancreatic lymph node showed metastatic involvement. Immunohistochemically, the tumor cells diffusely expressed Vimentin, S100 (Figure 4A), SOX10 (Figure 4B), CD56, and synaptophysin, while cytokeratin, EMA, HMB45 (Figure 4C), Melan-A (Figure 4D), CD117, DOG-1, and CD34 were all negative. The Ki-67 index was approximately 15% (the remaining immunohistochemical markers are shown in the Supplementary Figure S1). Fluorescence *in situ* hybridization (FISH) performed on paraffin-embedded tumor tissues confirmed the *EWSR1*-*ATF1* rearrangement using an EWSR1 break-apart probe (Figure 4E) and an *EWSR1*-*ATF1* dichromatic fusion probe (Figure 4F), respectively. The patient had no skin lesions or history of melanoma. Based on these findings, a final diagnosis of malignant gastrointestinal neuroectodermal tumor (M-GNET) in the body and tail of the pancreas was made. No adjuvant radiotherapy or chemotherapy was administered postoperatively. At the last follow-up (15 months), the patient was doing well and alive. The MRI results showed no signs suggestive of recurrence.

## Materials and methods

### Histopathology and immunohistochemistry

The tumor tissue was obtained from subtotal pancreatectomy/splenectomy performed at our institution and processed in our pathology department for histopathological evaluation and ancillary studies.

All surgically resected specimens were fixed in 10% neutral buffered formalin, routinely dehydrated, and embedded in paraffin. Paraffin blocks containing abundant tumor tissue were selected and serially sectioned at 4 μm, followed by staining with hematoxylin and eosin (H&E). Immunohistochemical staining was performed using commercially available antibodies according to the manufacturer’s instructions. Primary antibodies, purchased from Maixin Biotech (Fuzhou, China), included the following: Vimentin (MAB-0735), S100 (RAB-0150), SOX10 (RMA-0726), CD56 (MAB-0743), synaptophysin (MAB-0742), cytokeratin (Kit-0009), EMA (Kit-0011), HMB45 (MAB-0098), Melan-A (MAB-1033), CD117 (Kit-0029), DOG-1 (Kit-0035), CD34 (Kit-0004), CD10 (MAB-0668), ER (Kit-0012), *β*-catenin (RMA-1054), Desmin (MAB-0766), SMA (Kit-0006), STAT6 (RMA-0845), H3K27Me3 (RMB-0843), TLE1 (MAB-0686), and Ki67 (RMB-0731). The secondary antibody was applied using the MaxVision-HRP immunohistochemical kit (Kit-5030, Maixin Biotech, Fuzhou, China).

### FISH analysis

FISH analysis was performed on formalin-fixed, paraffin-embedded tissues using commercially available, locus-specific dual-color break-apart rearrangement probes. The EWSR1 break-apart probe (FP-051) and the EWSR1-ATF1 fusion probe (FP-081) were obtained from Healthcare-Bio (Wuhan, China). All procedures were carried out in following the manufacturer’s instructions, as previously described (6).

## Discussion

The question of whether CCSST and M-GNET constitute distinct tumor entities remains controversial. Some researchers propose that both tumors may originate from a common progenitor cell, as they share *EWSR1* gene rearrangements and the same *ATF1*/*CREB1* gene fusions at the molecular genetic level. The primary distinction lies in the expression of melanocytic differentiation markers: CCSST expresses HMB45, Melan-A, and MiTF, whereas M-GNET lacks these markers and does not exhibit melanosome precursors or melanosomes ultrastructurally. This difference likely reflects that tumors arising from primitive cells of neural crest/autonomic nervous system origin within the gastrointestinal tract retain primitive neuroectodermal/neural features in GNET. In contrast, CCSST follows a melanocytic differentiation pathway (5, 7–9). There are also differences in epidemiological and clinical prognostic features between CCSST and M-GNET. For example, in reports identifying the tumor as CCSST, the median patient age was higher, at 57 years (range: 35–85), and 84.6% of cases were male (10). In contrast, reports identifying the tumor M-GNET showed a median age of 33 years (range: 6–82), with no apparent sex predilection (11). Survival time did not differ significantly between the two groups, and both had poor prognoses. The median survival time for CCSST was 13.5 months, whereas for M-GNET, it was only 9.5 months (12–14). The presence of M-GNET over the extra-gastrointestinal sites, such as pancreas, may be explained by the impact of the genetic changes as well as the distinctive organ-specific microenvironmental niche for this rare tumor. Selective pressure and the ecological interactions among tumor cells, stromal elements and immune setting may exert influence on the phenotypic plasticity and clinical course of the tumor (15–17). This well warrants further investigation on ecology and evolution of rare tumor.

To our knowledge, CCSLTGT/M-GNET, an extremely rare soft tissue sarcoma, has never been reported in the pancreas. Reports of CCSLTGT/M-GNET occurring outside the gastrointestinal tract are also extremely rare, with only a few described in locations such as the tongue, neck and shoulder, esophagus, palate, bladder, bronchus, heart, etc. (11, 18–22). Interestingly, similar to CCSST, we observed a higher incidence in females than in males (F/M = 1.5:1), with a broad age range (5 to 82 years, mean age 39 years) (Table 2). In these cases, S100 was almost invariably staining positive (25/25). Additionally, 21 of the 25 cases were SOX10 nuclear positive, while 4 cases did not provide sufficient data. One case, originating from the tongue, was initially diagnosed as clear cell sarcoma, but the features were more consistent with M-GNET. Stockman et al. found that 86% of 14 cases exhibited *EWSR1* gene rearrangements, including *EWSR1::ATF* (50%) and *EWSR1::CREB1* (25%) (5). Among the 15 cases with confirmed *EWSR1-ATF1* gene fusions, 6 cases had *EWSR1-CREB1* fusions, 1 case (case 19) showed the rare *EWSR1-PBX1* fusion, 3 cases displayed *EWSR1* rearrangements without identified partner genes, and 1 case (case 4) had unavailable data. Morphologically, our case exhibited trabecular, cord-like, and nested arrangements, and immunophenotypically, the tumor cells were positive for S100 and negative for HMB45 and Melan-A—features consistent with M-GNET. From a molecular perspective, FISH confirmed an *EWSR1-ATF1* fusion, which aligns with the most frequently observed genetic alteration in M-GNET.

**Table 2:** Demographic of reported extra-enteric malignant gastrointestinal neuroectodermal tumors at present.

Summary	Value
Gender (male:female)	10:15
Median age, range (years)	39, 5–82
Location	
Skull base	1
Tongue	4
Palate	1
Larynx	1
Esophagus	3
Bronchus	1
Thigh	1
Heart	1
Retroperitoneum	1
Chest	1
Bladder	1
Neck and shoulder	4
Buttock and gluteus	2
Liver	1
Orbit	1
Pancreas (our case)	1
Total	25

M-GNET typically presents with nonspecific clinical manifestations. Most patients exhibit local symptoms, such as abdominal pain, intestinal obstruction, or an abdominal mass. A smaller subset may experience anorexia, weight loss, anemia, or systemic fever (5, 7). There is no significant difference in tumor size or location between M-GNET and CCSST, with the small intestine being the most commonly affected site (59%), followed by the colon (14.8%), stomach (13.9%), esophagus (4.1%), and other locations (8.2%) (5). Compared with CCSST, M-GNET arising in the gastrointestinal tract tends to show more aggressive biological behavior, with a shorter interval to first metastasis (1 month vs. 15 months) and an overall mortality rate ranging from 35 to 75%. As demonstrated by the lymph node metastasis observed in our case, approximately 50% of M-GNETs exhibit nodal involvement. While lymph node metastasis is exceedingly rare in sarcomas, both CCSST and M-GNET are exceptions. Therefore, accurate recognition and diagnosis of this neoplasm by pathologists is critically important, as it helps clinicians in determining whether lymph node dissection is necessary.

Given that the case occurred in the pancreas and was characterized by spindle and epithelioid cells with clear cytoplasm, our differential diagnoses mainly included primary or metastatic malignant melanoma, perivascular epithelioid cell tumor (PEComa), solid pseudopapillary neoplasm (SPN) of the pancreas, pancreatic neuroendocrine tumor (PanNEN), and gastrointestinal stromal tumor (GIST). Morphologically, malignant melanoma can closely resemble CCSST/M-GNET. Therefore, a history or presence of cutaneous lesions is crucial, as melanocytic features on histology are indicative. In addition to being positive for SOX10 and S-100, melanoma typically expresses HMB45, Melan-A, and MiTF. Molecular testing may also reveal *BRAF (V600E)* or *c-Kit* mutations. However, it is important to note that sarcomatoid melanoma may lose expression of HMB45 and Melan-A, making molecular testing particularly valuable for diagnostic confirmation. PEComas in the pancreas are exceedingly rare, typically occurring in females. While their microscopic appearance may overlap with M-GNET, PEComa tumor cells characteristically express smooth muscle markers (e.g., SMA) and melanocytic markers (HMB45 and Melan-A). These markers, however, are notably absent in M-GNET. SPN of the pancreas is a low-grade malignant epithelial tumor more prevalent in young women. It forms solid nests, trabeculae, and pseudopapillary structures. SPN typically expresses vimentin, LEF1, *β*-catenin, and synaptophysin, and molecular studies show that most SPNs harbor CTNNB1 exon 3 mutations. Grade 1 or 2 PanNENs often display organoid growth patterns, including solid nests, trabeculae, and acinar formations. The tumor cells are relatively uniform with mild pleomorphism, round to oval nuclei, and fine chromatin, although variants with clear, eosinophilic, or pleomorphic cells may occur. Diffuse positivity for synaptophysin and chromogranin A helps confirm neuroendocrine differentiation. Primary GISTs arising in the pancreas are rarely reported, whereas GISTs originating from the stomach or duodenum that secondarily involve the pancreas are more common. GISTs exhibit spindle-shaped, epithelioid, or mixed cellular morphologies. They characteristically express CD117, DOG1, and CD34, and molecular testing frequently reveals *c-Kit* or *PDGFRα* mutations. Notably, *EWSR1* gene rearrangements are absent in GISTs, which helps differentiate them from M-GNET.

Treatment for M-GNET primarily involves surgical resection, often accompanied by regional lymph node dissection. In some cases, c-Met/ALK inhibitors and VEGF inhibitors may also be considered. A few patients have shown partial responses to anlotinib and apatinib (23, 24). However, due to the rarity of this tumor, no standardized therapeutic regimen currently exists. In the case of our patient, no adjuvant radiotherapy or chemotherapy was administered postoperatively. Follow-up imaging revealed no evidence of recurrence, suggesting a relatively indolent biological behavior in this case. M-GNET is an established entity of the gastrointestinal tract but yet is a rare tumor in the pancreas. Since the tumor is rarely found in pancreatic tissue, this feature can be faced as a diagnostic challenge, therefore, the information about its clinico-pathological and molecular features is important and this case report would contribute to the near understanding of M-GNET. This manuscript presents a rare case of primary pancreatic malignant gastrointestinal neuroectodermal tumor, which is a diagnostically challenging neoplasm. Although this case is the reported in the pancreas, previous cases of M-GNET have been documented in other locations, including the gastrointestinal tract (25). By integrating morphological features, immunohistochemical profiles, and molecular findings, a definitive diagnosis can typically be made. However, it remains uncertain whether there are additional, yet-undiscovered pathological features unique to pancreatic M-GNET, and continued follow-up is necessary to assess the patient’s long-term prognosis. A more comprehensive understanding of M-GNET occurring at extragastrointestinal sites is urgently needed.

## Data Availability

The original contributions presented in the study are included in the article/supplementary material, further inquiries can be directed to the corresponding authors.
