# Supplementary material for: A rare case of clear cell sarcoma-like/malignant gastrointestinal neuroectodermal tumor in the pancreas: case report and literature review
Source: Front Med (Lausanne). 2026 Mar 19;13:1768345. doi: 10.3389/fmed.2026.1768345 (PMC13044776; doi:10.3389/fmed.2026.1768345)
Supplement: Supplementary file 1 [file Image_1.pdf]

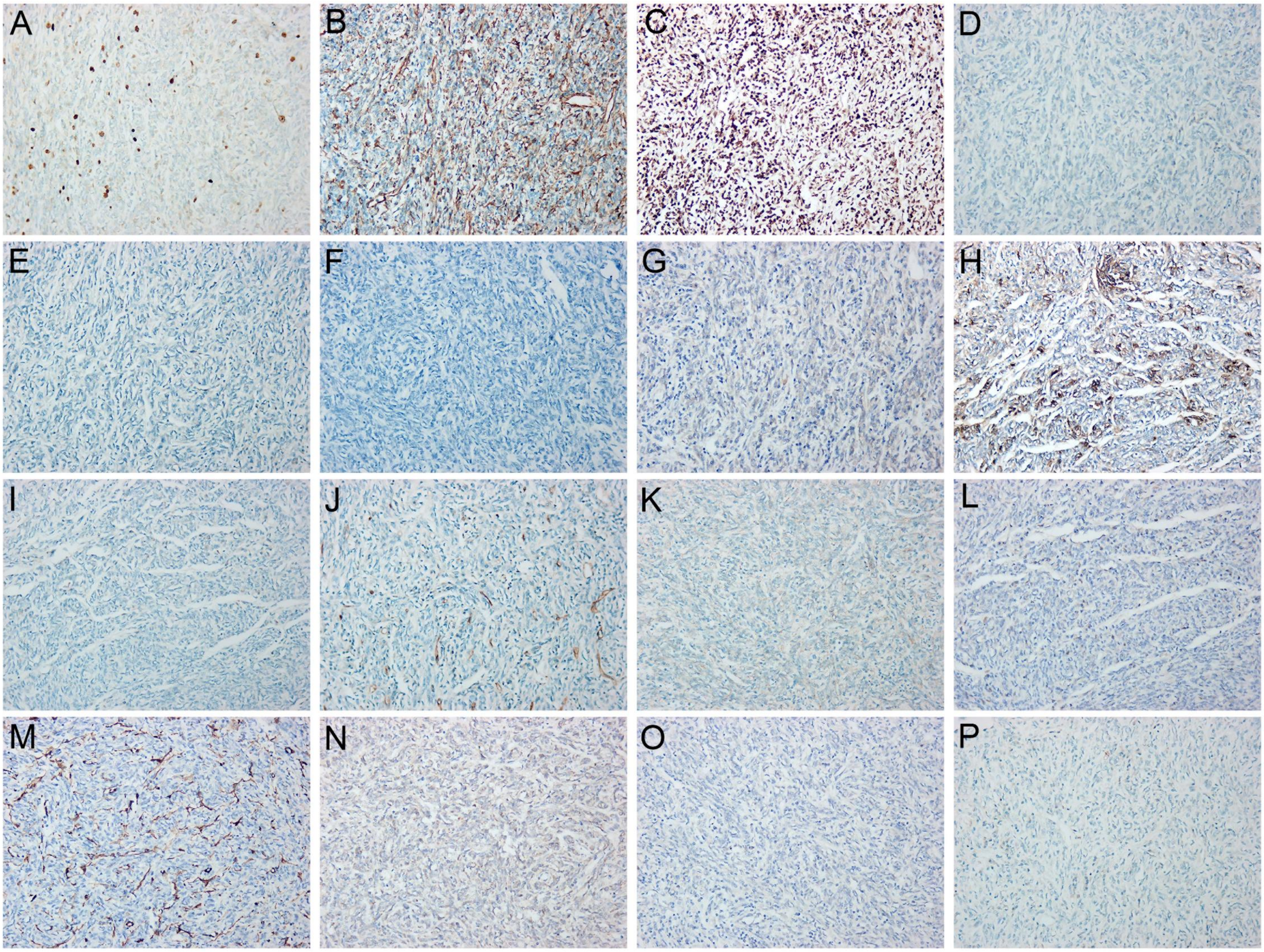

Supplementary Figure. S1

(A)Ki-67 index was approximately 15%, 200x

(B)Vimentin expression was variable. Note staining of neoplastic cells arranged in nests surrounded by desmoplastic stroma, 200x

(C) H3K27Me3 nuclear positive, no expression loss, 200x

(D) CD117 negativity, 200x

(E) Pan-cytokeratin negativity, 200x

(F) Desmin negativity, 200x

(G) DOG1 negativity, 200x

(H) EMA expression was variable, focal area positive, 200x

(I) ER negativity, 200x

(J) CD34 negativity, 200x

(K) β-catenin negativity, 200x

(L) MDM2 negativity, 200x

(M) SMA negativity, 200x

(N) STAT6 negativity, 200x

(O) TLE1 negativity, 200x

(P) DOG1 negativity, 200x
